# IQN path ASBL report from the first European cfDNA consensus meeting: expert opinion on the minimal requirements for clinical ctDNA testing

**DOI:** 10.1007/s00428-019-02571-3

**Published:** 2019-04-26

**Authors:** Zandra C. Deans, Rachel Butler, Melanie Cheetham, Elisabeth M. C. Dequeker, Jennifer A. Fairley, Francesca Fenizia, Jacqueline A. Hall, Cleo Keppens, Nicola Normanno, Ed Schuuring, Simon J. Patton

**Affiliations:** 1 UK NEQAS for Molecular Genetics, Department of Laboratory Medicine, Royal Infirmary of Edinburgh, Little France Crescent, Edinburgh, EH16 4SA UK; 2 All Wales Genetic Laboratory, Institute of Medical Genetics, University Hospital of Wales, Heath Park, Cardiff, CF14 4XW UK; 3 European Molecular Genetics Quality Network, Manchester Centre for Genomic Medicine, St Mary’s Hospital, Manchester, M13 9WL UK; 4 Biomedical Quality Assurance Research Unit, Department of Public Health and Primary Care, KU Leuven, Leuven, Belgium; 5 University Hospital of Leuven, Leuven, Belgium; 6 Cell Biology and Biotherapy Unit, Istituto Nazionale Tumori “Fondazione G. Pascale”-IRCCS, Naples, Italy; 7 International Quality Network for Pathology (IQN Path ASBL), 3A Sentier de l’Esperance, 1474 Luxembourg, Luxembourg; 8 Department of Pathology, University of Groningen, University Medical Center of Groningen, Groningen, The Netherlands

**Keywords:** ctDNA, cfDNA, Liquid biopsy testing

## Abstract

Liquid biopsy testing is a new laboratory-based method that detects tumour mutations in circulating free DNA (cfDNA) derived from minimally invasive blood sampling techniques. Recognising the significance for clinical testing, in 2017, IQN Path provided external quality assessment for liquid biopsy testing. Representatives of those participating laboratories were invited to attend a workshop to discuss the findings and how to achieve quality implementation of cfDNA testing in the clinical setting, the discussion and outcomes of this consensus meeting are described below. Predictive molecular profiling using tumour tissue in order to select cancer patients eligible for targeted therapy is now routine in diagnostic pathology. If insufficient tumour tissue material is available, in some circumstances, recent European Medicines Agency (EMA) guidance recommends mutation testing with plasma cfDNA. Clinical applications of cfDNA include treatment selection based on clinically relevant mutations derived from pre-treatment samples and the detection of resistant mutations upon progression of the disease. In order to identify tumour-related mutations in amongst other nucleic acid material found in plasma samples, highly sensitive laboratory methods are needed. In the workshop, we discussed the variable approaches taken with regard to cfDNA extraction methods, the tests, and considered the impact of false-negative test results. We explored the lack of standardisation of complex testing procedures ranging from plasma collection, transport, processing and storage, cfDNA extraction, and mutation analysis, to interpretation and reporting of results. We will also address the current status of clinical validation and clinical utility, and its use in current diagnosis. This workshop revealed a need for guidelines on with standardised procedures for clinical cfDNA testing and reporting, and a requirement for cfDNA-based external quality assessment programs.

## Introduction

The National Cancer Institute defines liquid biopsy as a test performed on blood samples in order to look for cancer cells from a tumour that are circulating in the blood, or for pieces of DNA from tumour cells that are in the blood [1]. Being a non-invasive, cost-effective procedure, which rapidly detects and monitors molecular biomarkers in cancer patients, analysis of plasma cfDNA testing has shown great promise to become a useful clinical tool. It is particularly useful in identifying targeted treatments and monitoring tumour response to therapy.

However, plasma cfDNA testing is not without challenges: establishment of optimal timings between blood draw and blood processing, optimising DNA extraction procedures, identifying appropriate analysis methods and ensuring accurate interpretation of results. With these issues in mind, IQN Path, a network of quality assessment experts with an interest in cancer biomarker testing, collected data on liquid biopsy testing in Europe in 2017 [2] provided an external quality assessment [3] and organised a cfDNA workshop which was attended by European key opinion leaders. The purpose of the workshop was to summarise the current limited knowledge on liquid biopsy testing and to establish a white paper to emphasise the need for standardisation of the implementation cfDNA testing in clinical practice. This article summarises the workshop discussions.

## Current applications of cfDNA mutation testing

The current clinical applications of cfDNA mutation testing are the identification targeted therapies in non-small cell lung cancer (NSCLC) by testing for epidermal growth factor receptor (*EGFR)* mutations, and the assessment of Kirsten RAt Sarcoma (*KRAS), Neuroblastoma RAS viral oncogene homologue (NRAS)* and v-Raf murine sarcoma viral oncogene homologue B (*BRAF)* mutation status in patients with colorectal cancer (CRC), in situations when molecular testing is not feasible on tissue due to insufficient/inadequate material or inability to perform a biopsy [4]. Clinical trials have shown that analysis of serial cfDNA samples during cancer treatment can monitor tumour response and identify early emergence of any resistance mechanisms [5–8]. Following targeted therapy, cfDNA testing can identify treatment-resistant mutations. In the event that cfDNA plasma testing does not detect a mutation, a solid tumour tissue biopsy and the cfDNA plasma test should be conducted concurrently, as the results may help inform recommendations for novel treatment options.

Different tumour types raise their own particular issues [9]. For example, in patients with NSCLC who have brain metastases or intra-thoracic disease, the chances of detecting cfDNA mutations in plasma are reduced [10]. In patients with gastrointestinal stromal tumours (GIST), targetable *KIT* mutations in pre-treatment tumour biopsies are not detected in one in nine patients with localised or local advanced disease, whereas in 13 of 14 cases with metastasized advanced GIST, *KIT* mutations are detected in pre-treatment plasma [6]. In patients with CRC [7, 11–13] and with NSCLC regarding the detection of resistance mutations upon progression on tyrosine kinase inhibitors [14, 15], a key issue is the sensitivity of the different assays.

Today, the clinical utility of cfDNA testing that is required to evaluate whether clinical outcome for patients who were treated based on the test has improved compared to those not tested, and has not been performed for most tumour types despite major achievements regarding analytical validity and clinical validity [16, 17]. Therefore, cfDNA testing is not appropriate for diagnosis (without tissue diagnosis). The only two FDA-approved cfDNA-based tests with clinical utility are the cobas *EGFR* Mutation Test v2 (Roche Diagnostics) detecting *EGFR* mutations in cfDNA from patients with lung cancer [3, 18] and the Epi proColon assay (Epigenomics AG) for the detection of SEPT9 promoter methylation in cfDNA from patients undergoing screening for CRC [19].

However, the expectations of the clinical applications of cfDNA testing are high. If implemented well, performed at high quality (according to ISO 15189) [20], it can be a reliable, robust, reproducible, cost-effective and accurate test with a fast turn-around time. It is anticipated that in the future, a trend towards multiplexed and quantitative cfDNA testing methods as these will yield even larger amounts of useful clinical information.

## Sample collection and processing

The volume of blood required for cfDNA analysis is dependent upon the testing methodology but generally ranges between 6 and 10 ml. Clearly, handling of blood samples impact on the quality of the cfDNA testing and on the downstream results and tubes should be spun and processed as soon as possible. Therefore, tubes selected for blood collection must be appropriate to maintain the integrity of the sample, taking into consideration the time between the blood draw and laboratory processing, the availability of any storage facilities and the mechanism of transport to the processing laboratory. Current methods typically involve blood collection in EDTA anti-coagulant tubes, storage at 4 °C, and transportation to the pathology laboratory where the sample is processed and stored at −80 °C, all within a timeframe of 6 h. However, these options are not always feasible; therefore, the use of tubes containing preservatives to prevent haemolysis and to reduce the degradation of cfDNA is becoming increasingly common practice to allow an extended period of time before blood must be processed. Laboratories must apply acceptance criteria to ensure that received samples are suitable for cfDNA mutation testing. Processing protocols should entail a double centrifugation protocol, including initial slow centrifugation, with the eluted plasma subjected to a second fast centrifugation. The resultant plasma layer can be used for cfDNA extraction immediately or can be stored either at − 20 °C for fewer than 5 days or at − 80 °C for longer storage. Freeze thawing sample aliquots is not recommended. Table 1 summarises the advantages and disadvantages of current options.

**Table 1:** Summary of the advantages and disadvantages of available blood collection tubes

Tube type	Examples	Advantages	Disadvantages
EDTA anti-coagulant	• EDTA K_2_ • EDTA K_3_	• Inexpensive • Readily available • Follows standard processes for handling of blood tubes	• Processing within 6 h
Preservatives	• StreckCell-free DNA BCT • Qiagen PAXgene Blood ccfDNA tubes • Roche cFDNA tubes • ThermoFisher Liquid Biopsy Blood collection kit • Norgen cfDNA Preservation tube • MagBio Blood STASIS 21ccfDNA tubes • Biomatrica LBgard blood tubes • CellSave Preservation tubes (Janssen Diagnostics)	• Storage and transport at ambient temperature • Provides up to 72 h for processing • Readily available	• Expensive • Out of step with usual practice for handling of blood tubes i.e. storage at ambient temperature • Some only available as a glass tube

## DNA extraction

Plasma samples can contain a variable amount of high molecular weight (HMW) DNA as a result of haemolysis during processing. In contrast, plasma ctDNA (circulating tumour) fragments have an average size of 132–1450 base pairs (bp) corresponding to mononucleosome-protected DNA [17, 21]. With growing interest in cfDNA-based diagnostics, a number of cfDNA-focused extraction kits are available from various manufacturers (Table 2). However, none of the current cfDNA extraction methodologies enrich either ctDNA or the nucleosome-protected DNA fragments. Studies comparing cfDNA extraction methods and kits revealed large differences in total DNA yield [22–24]. These findings may be due to a variety of reasons, including variations in extraction methodology, plasma input and elution volume, or that with low volumes of available plasma cfDNA, there is a tendency to load larger plasma volumes and elute the cfDNA fragment with the lowest volume to maximise concentration. So, to select the optimal cfDNA extraction kit, factors such as the extraction method, time, throughput and price should be considered.

**Table 2:** Examples of currently available cfDNA extraction methods

cfDNA extraction kit	Manufacturer
QIAamp® circulating Nucleic Acid Kit	Qiagen
QIAamp® DNA Blood Mini Kit	Qiagen
EZ1® ccfDNA Kit	Qiagen
QIAsymphony PAXgene Blood ccfDNA Kit	Qiagen
QIAsymphony DSP Circulating DNA Kit	Qiagen
QIAamp® MinElute® ccfDNA	Qiagen
cfDNA isolatie van Quick-cfDNA™ Serum and Plasma DNA Miniprep Kit	Zymo Research
Maxwell® RSC ccf Plasma Kit	Promega
Cobas® cfDNA Sample Preparation Kit	Roche Diagnostics
NucleoSpin® Kit	Macherey-Nagel
MagNA Pure Isolation System	Roche Diagnostics
Plasma ccf DNA purification kit	Norgen BioTek
Chemagic cfDNA isolation kit	Perkin-Elmer
FitAmp Plasma/Serum DNA isolation kit	Epigentek
PME free circulating DNA extraction KIT	Analytik Jena
EpiQuick™ cfDNA Isolation Easy Kit	Epigentek
NEXTprep-Mag™ cfDNA Kit	Bio Scientific/PerkinElmer
Idylla™ ctKRAS Plasma Test (DNA extraction is part of detection-test)	Biocartis

To compare the performance of different cfDNA extraction methods, most studies focus on cfDNA yield, quantifying the yield with techniques such as fluorospectroscopy, fluorometry and quantitative real-time PCR (qPCR). In general, quantification of DNA elutes shows a significant correlation with qPCR [23], while non-double-stranded DNA measurement assays do not.

High levels of nucleosome-protected DNA from non-tumour tissues in addition to HMW DNA can complicate ctDNA molecular analysis by increasing the rate of false-negatives. Hence, the integrity of any extracted cfDNA should be tested. However, typical methods that measure cfDNA quantity do not assess extracted DNA integrity. Integrity testing options include fragment analysis which employs capillary electrophoresis to determine DNA fragment length: The size of DNA fragments is used to evaluate the relative amount of nucleosome-protected 140–160 bp DNA fragments compared to HMW DNA and DNA degradation [22, 23]. More recently, amplifiable DNA concentrations and the fragment size have been measured using very small amounts of cfDNA in various single-tube multiplex digital droplet PCR (ddPCR) assays [6, 25, 26].

So, in the case of insufficient cfDNA, the cfDNA should be quantified using a double-stranded DNA method before proceeding to PCR or sequencing analysis. In addition, as various factors can complicate the molecular analysis of cfDNA, the integrity of any extracted cfDNA should be estimated. Finally, the amount of ctDNA can not only be influenced by pre-analytical procedures such as haemolysis, centrifugation and cfDNA extraction procedures, but it has been reported that ctDNA levels differ significantly between various tumour types, stages of disease, tumour volume and stages of treatment [6, 9]. Different levels of total cfDNA are also influenced upon extreme exercise, infectious disease and age [27]. In short, we should realise that today, the dynamics of the release of DNA from cancer cells into the circulation, the mechanism of clearance as well as half-life of cfDNA, are still poorly understood.

## Testing methods

Plasma often contains very low levels of cfDNA; therefore, methods are needed, different from those used for targeted detection of defined oncogenic variants in tissue biopsy, with a very high analytical sensitivity (0.01–0.1%). Assays for the detection of the same variants in cfDNA include amplified refractory mutation system (ARMS), allele-specific quantitative PCR, PCR with peptide nucleic acid clamps, next-generation sequencing (NGS), BEAMing and ddPCR [5, 17, 28, 29]. These assays should be optimised for sensitivity in order to detect cfDNA which may be present in lower concentrations than DNA isolated from cancer tissue [30]. Many laboratories have focused on improving cfDNA assay specificity, in order to make it more useful as a screening tool. A meta-analysis of 20 studies demonstrated that specificities achieved using cfDNA were comparable to solid tissue genotyping [31]. The performance of NGS, which enables broader gene profiling, has also been evaluated using cfDNA with some studies showing similar sensitivities and specificities when compared to single-gene assays [32–35]. In contrast, an international pilot External Quality Assessment (EQA) scheme observed a higher method-specific error rate for NGS compared to ddPCR and commercial kits [3].

The choice of assay will depend on multiple factors including clinical requirements, throughput, specificity, sensitivity, and access to equipment, expertise and budget. Table 3 summarises some of the commonly used assays for detecting clinical-relevant *EGFR* variants in cfDNA derived from NSCLC. The clinical oncology demand for testing of variants other than *EGFR* will help establish multigene testing as routine practise in future.

**Table 3:** Examples of assays used to detect common *EGFR* mutations in ctDNA extracted from cell-free plasma discussed in the workshop. This is not an exhaustive list of possible methods but a summary of some approaches currently in use or discussed by the workshop participants. There are other options available such as various NGS-platforms with panels (e.g. Illumina TruSeq), RT-PCR-based assays (e.g. Diatech Pharmacogenetics Easy® *EGFR* kit). As conflicting information was available from workshop participants and discussed at the workshop, then no data has been supplied for minimum amount of DNA required

Test	Therascreen EGFR Plasma RGQ PCR Kit	Droplet digital PCR	Oncomine™ Lung cfDNA Assay	OncoBeam™ EGFR Kit	cobas® EGFR Mutation Test
Version	1	Not applicable	1	1	2
Manufacturer	QIAGEN	BioRad	ThermoFisher	Sysmex Inostics	Roche Molecular Diagnostics
CE marked?	Yes	No	No	No	Yes
FDA approved?	No	No	No	No	Yes
Plasma specific assay?	Yes	No but appropriate to use	Yes	Yes	Yes
Assay type	Real time PCR (ARMS and Scorpions)	Digital PCR (nanoparticle)	CDx using Ion Torrent™ Next-Generation Sequencing	Digital PCR (BEAMing)	Real time PCR (cobas z4800 analyser)
Strategy	Targeted mutations in the *EGFR* gene including p.(T790M), p.(L858R) and 12 different deletions in exon 19	Targeted mutations with specific designed BioRad kits for most *EGFR, BRAF* and *KRAS* variants. Amongst other options for custom design	Targeted panel: 11 genes (*ALK, BRAF, EGFR, ERBB2, KRAS, MAP2K1, MET, NRAS, PIK3CA*, *ROS1* and *TP53*), and > 150 hotspots	Targeted mutations in the EGFR gene including p.(T790 M), p.(L858R), p.(C797S), and five different deletions in exon 19	Targeted mutations in the *EGFR* gene including p.(T790M), p.(L858R), p.(G719X), p.(S768I), p.(L861Q), five different insertions in exon 20 and 29 different deletions in exon 19
Testing TAT (days)	< 1	< 2	2–3	10	< 1
Analytical sensitivity	Mutation specific (*C*_t_ ≤ 7.40–8.90)	High sensitivity	High sensitivity	High sensitivity	High sensitivity
Limit of detection (LOD)^a^	Mutation specific (0.8–17.5%)	0.01% depending on DNA input	0.1% (20 ng input cfDNA)	0.04% with 2 ml plasma	Mutation specific (10–100 copies mutant DNA/ml plasma)
Advantages	Easy to use Fast (< 1 day). Tests for p.T790M	High analytical sensitivity Fully quantitative Also suitable with minimal amounts of cfDNA Custom design ddPCR for any mutation	Multiplexed—many different mutations High analytical sensitivity	High analytical sensitivity Quantitative Tests for p.(T790 M)	Easy to use Fast (< 1 day) Tests for p.(T790M)
Disadvantages	High input DNA requirement Requires dedicated RT PCR platform (Rotor-Gene Q) Poor analytical sensitivity (most mutations > 2.5%)	Requires dedicated and well-trained personnel Labour intensive	High input DNA requirement Requires dedicated and well-trained personnel. Requires expensive equipment. Labour intensive Slow TAT	Complex to set up and use Low throughput slow TAT	High input plasma requirement. Semi quantitative assay
Comments	Not recommended for monitoring due to high DNA requirement	Recommended for monitoring because of quantitative nature of the test	Recommended for predictive testing		FDA-approved for detection of *EGFR*-T790M mutation upon progression on EGFR-TKI
References	www.qiagen.com	www.bio-rad.com	www.thermofisher.com	www.sysmex-inostics.com	https://molecular.roche.com/

^a^Information as described in the manufacturer’s information

## Reporting results

Reporting the results of plasma cfDNA mutation testing should follow standard guidelines for reporting of molecular pathology results [36, 37] and adhere to the ISO15189 standard for medical laboratories [20]. Reports should be clear and concise within a maximum of two pages. It should be clearly stated that cfDNA testing has been performed and include two patient-specific identifiers, a sample identifier and the sample type tested. Pagination should be used and included with the date and time of sampling, a clear statement of the results, details of the testing method performed, limitations of the test including sensitivity of the assay, reason for referral, appropriate interpretation of results and details of the reporter and authoriser. Genotyping results should be given according to Human Genome Variation Society (HGVS) nomenclature [38], and the predicted protein change should be presented with appropriate reference sequences.

Further details may be included if it pertains to the result, for example, the tube type used for blood collection or if there is evidence of haemolysis. These details should be noted within the molecular testing laboratory even if not included on the external report.

The sensitivity of the method of cfDNA analysis should be stated. As the total amount of cfDNA will vary, it is recommended that both the variant allele frequency (VAF) and the amount of mutant copies per milliliter of plasma are reported. Standardisation is encouraged to aid result interpretation and for the comparison of data between laboratories. Ultimately, the total cfDNA prior to the analysis of mutations and the limit of sensitivity of the assay are useful additions to any reporting. Any previous molecular analyses should also be outlined and previously detected mutations re-stated, for subsequent analysis may include the detection of primary mutations.

The interpretation of the molecular result depends on the gene analysed, the tumour type and the clinical context of the gene-drug interaction. However, the analysis of cfDNA requires additional consideration, as the presence or absence of cfDNA is unknown in the absence of a detectable mutation. The detection of a mutation will generally lead to a simple interpretation of the mutation present result. However, a mutation absent result could be due to a variety of reasons including:

- The mutation is not detected and is therefore a true negative.
- Insufficient sample of ctDNA giving a possible false negative.
- The sampling method destroyed any ctDNA giving a possible false negative.
- The analytical method is insufficiently sensitive to detect low levels of mutant ctDNA, resulting in a false negative.

When no mutation is detected, the report should either include a remark that “mutation not detected does not rule out the presence of a mutation because the analysis depends on LOD of the test, and quality and quantity of the input cfDNA” or contain specific information on the quality control metrics of the assays regarding the LOD of the tested sample. For example, for ddPCR, this should be based on the number of mutant droplets in no template controls divided by the total number of positive droplets. A “mutation absent” result must be treated with caution, and the above possibilities should be referred to, and repeat sampling recommended, preferably by biopsy. Dependent on the gene, tumour type and clinical context, additional controls may be included which may aid interpretation, as previously described.

## Quality considerations

High-quality cfDNA testing procedures yield high quality results. Validation and verification of laboratory methods and procedures ensure a safe and useful service for clinicians and patients. However, errors in the pre-analytical phase can lead to false-positive or false-negative results which can result in misdiagnosis and inappropriate treatment. There are a number of issues to consider in the establishment and maintenance of high-quality processes.

Laboratories must have sample acceptance criteria which are then communicated to the clinicians requesting and collecting samples. Sample storage must be documented and monitored, and further sample preparation procedures validated. Methods should be optimised for sensitivity and cost-effectiveness. Test development, assessment of utility and performance specification should be properly documented.

Test validation should include different kinds of variants such as point mutations or small insertions and deletions, as well as positive and negative internal quality controls, the type of which largely depends on the methodology used. Validation should focus on samples that have been processed through all clinical workflows, and critically on cfDNA samples with variants at the limits of sensitivity. In addition, to permit the reliable detection of rare clinically relevant variants, regular external and internal validation should assess and monitor the quality of test results and, when required, modify existing methods and procedures.

Commercially available reference standards can be used as internal quality controls. To monitor the performance of each test, it is recommended that samples harbouring a different percentage of mutant allele up to the limit of detection are used. However, it should be noted that cell line and synthetic materials may not be optimal substitutes for ctDNA derived from blood or plasma [39].

Laboratories are advised to optimise their workflow design and embrace quality control parameters. To avoid contamination, different test phases should be performed in contained compartments. Turnaround time should be closely monitored, and action taken the clinically acceptable timeframe is exceeded. Detailed standard operating procedures are required, and all personnel should be adequately trained in these and kept informed of any changes. Laboratories are encouraged to regularly demonstrate that their testing procedures deliver accurate and reliable results by implementing regular internal audits and to participate in external quality assurance. We strongly recommend that all processing steps should be performed and monitored according to the requirements of ISO15189 [20].

Finally, when outsourcing any part of cfDNA analysis to an external laboratory, the referring laboratory must be responsible for documenting procedures used for the selection and evaluation of the external laboratory and should monitor the quality of performance and ensure that it is competent to perform the required testing.

## Conclusion

It was clear from the workshop that many laboratories in Europe already perform cfDNA testing or are planning to adopt it for NSCLC for treatment selection and resistance monitoring. This paper summarises the discussions about cfDNA testing in clinical practice from the workshop and encompasses several aspects of cfDNA mutation testing, including technical considerations, quantity and quality of extracted cfDNA, the advantages and disadvantages of different testing methods, reporting of results and quality assurance and quality control. The workshop highlighted the different approaches taken by testing laboratories through the pathway, end to end, i.e. from blood collection, cfDNA extraction method, assays performed and reporting of the results. cfDNA testing has the potential for broader applications in clinical practice in the future, and we anticipate that the use of multiplex methods will continue to increase as larger volumes of data can be generated from one sample that may provide additional insights into treatment options for patients.
